# Multifactorial prevention program for cardiovascular disease in primary care: hypertension status and effect on mortality

**DOI:** 10.1038/s41371-024-00900-x

**Published:** 2024-02-20

**Authors:** Susanna M. Kuneinen, Hannu Kautiainen, Mikael O. Ekblad, Päivi E. Korhonen

**Affiliations:** 1 Department of General Practice, University of Turku and Turku University Hospital, Turku, Finland; 2 Pihlajalinna Plc, Turku, Finland; 3 Folkhälsan Research Center, Helsinki, Finland; 4 Unit of Primary Health Care, Kuopio University Hospital, Kuopio, Finland

**Keywords:** Preventive medicine, Diagnosis

## Abstract

The aim of this study was to investigate if mortality during a 13-year follow-up varied between normotensive subjects, screen-detected hypertensive subjects, and subjects with antihypertensive medication at baseline. A population-based screening and intervention program identified 2659 apparently healthy, middle-aged cardiovascular-risk persons in southwestern Finland. Screen-detected hypertension was verified by home blood pressure measurements. Lifestyle counseling was provided for all participants and preventive medications were started or intensified if needed. All-cause and cardiovascular mortality were obtained from the official statistics. Screen-detected hypertension was diagnosed in 17% of the participants, 51% were normotensive and 32% had antihypertensive medication at baseline. The screen-detected hypertensives had higher mean blood pressure and cholesterol levels than the two other groups. Altogether 289 subjects died during the follow-up, 83 (29%) from cardiovascular disease. Those with screen-detected hypertension had decreased cardiovascular mortality risk compared to the medicated hypertensives [sHR 0.40 (95% CI: 0.19 to 0.88, *p* = 0.023)], and comparable with that of the normotensives [sHR 0.53 (95% CI: 0.24 to 1.15)]. Newly diagnosed diabetes at baseline was a powerful predictor of cardiovascular mortality [sHR 2.71 (95% CI: 1.57 to 4.69)]. Early detection of hypertension and timely multifactorial intervention seem to be important in preventing hypertension-related mortality.

## Introduction

Hypertension is the most important, but treatable risk factor for cardiovascular disease (CVD) and death worldwide, leading to 10.5 million deaths each year [1, 2]. Hypertension has been estimated to account for approximately 50% of all heart disease and stroke-related deaths [3], which together are the biggest causes of morbidity and mortality globally [1, 2]. Over the past 25 years, the number of people with elevated blood pressure (BP) has increased significantly [1]. The increasing prevalence of hypertension is attributed to population growth, ageing, and behavioral factors, such as unhealthy diet, harmful alcohol consumption, physical inactivity and excess weight [3].

The diagnosis of hypertension is fairly easy by the at-home BP measurements, and effective low-cost medications are widely available. Also, the benefits of treating hypertension to conventional BP levels, e.g., less than 140/90 mmHg, for primary prevention of CVD have been well established [4–6]. Nevertheless, hypertension remains commonly undetected and undermedicated. It has been estimated that untreated or inadequately treated hypertension concerns approximately 20% of the adult population globally [7]. The World Heart Federation and the World Health Organization (WHO) advocate the importance of improving awareness and early detection of raised BP and highlight the importance of both high-risk and population-based strategies in hypertension management and control [3, 8]. However, high-certainty evidence about the effectiveness of mass, targeted, or opportunistic screening strategies for reducing morbidity and mortality associated with hypertension is lacking [9].

In this prospective cohort study, we examined the impact of a community-based cardiovascular risk factor screening and intervention program [10] on mortality. Specifically, we sought to investigate if the risk of death varied between the subjects with screen-detected hypertension, the subjects with antihypertensive medication prior to screening, and the subjects who were normotensive at baseline. We now report the findings on mortality after 13 years of follow-up.

## Materials and methods

### Subjects

Men and women aged 45–70 years living in the semirural Finnish towns of Harjavalta and Kokemäki (6013 eligible inhabitants on 31.12.2007) were invited to participate in the Harmonica (Harjavalta Risk Monitoring for Cardiovascular Disease) Project. Institutionalized persons and individuals with previously diagnosed CVD or diabetes were excluded from this primary prevention project. Screening and interventions were performed from August 2005 to September 2007.

The study procedures have been described in detail previously [10]. Briefly, a cardiovascular risk factor survey, a tape for the measurement of waist circumference (WC), and a type 2 diabetes (T2D) risk assessment questionnaire (FINDRISC, Finnish Diabetes Risk Score, available from www.diabetes.fi/english) [11] were mailed to every eligible inhabitant. In the risk factor survey, subjects were asked to measure their WC at the level of umbilicus (inclusion criteria: WC ≥ 80 cm in women and ≥94 cm in men in Harjavalta), to report the latest measured BP (inclusion criteria: BP ≥ 140/90 mmHg), their use of antihypertensive medication, their history of gestational diabetes or hypertension, and history of coronary heart disease, myocardial infarction, or stroke of their parents or siblings. The subjects were asked to fill-in and mail the risk factor survey to the public health care center if they were willing to participate in the project. Participation and all the measurements included were free of charge for the subjects. The response rate as 74% (4450 of 6013).

The respondents with at least one above-mentioned risk factor or ≥15 points (≥12 points in Harjavalta) in the FINDRISC were invited for laboratory tests and an appointment with a trained public health nurse. The stringent inclusion criteria regarding WC and FINDRISC scores were used in Kokemäki for logistical reasons and due to limited financial resources.

### Appointment with the study nurse

Waiting for the nurse’s appointment, the study participants completed self-administrated questionnaires including details on education, current smoking, alcohol consumption (Alcohol Use Disorders Identification Test, AUDIT [12]) and leisure-time physical activity (LTPA). At the nurse’s appointment a physical examination (including measurements of BP, WC, height and weight) was performed and lifestyle counseling was given [10]. The presence of ongoing antihypertensive medication was confirmed from the patient and from the medical records. Subjects with hypertension, newly detected glucose disorders, metabolic syndrome (MetS), obesity, or ≥5% risk for developing a fatal CVD event according to the Systematic Coronary Risk Evaluation System (SCORE) [11] were categorized as high-risk subjects, and they were offered to have an appointment with the general practitioner (GP) of the project. The persons who attended the nurse’s or the nurse’s and GP’s appointment, were included in the present study (*n* = 2659).

### Measurements

BP was measured with a calibrated mercury sphygmomanometer by a trained nurse with subjects in a sitting posture after resting for at least five minutes with the cuff placed on the arm. An appropriate-sized cuff was used, depending on the circumference of the arm. In each participant, the mean of two BP readings taken at intervals of at least two minutes was used in the study. Subjects were provided with and taught to use an automatic validated BP device (Omron® M4-1, the Netherlands) for home BP monitoring, if the nurse measured the mean systolic BP ≥ 140 mmHg or the mean diastolic BP ≥ 90 mmHg and the subject did not have ongoing antihypertensive medication. These subjects were instructed to take duplicate BP measurements at home after five minutes of rest in the morning and evening for one week. The mean home BP was calculated from the recorded measurements excluding the first day [13].

Body mass index (BMI) was calculated as weight (kg) divided by the square of height (m²). MetS was defined according to the criteria of the International Diabetes Federation (IDF) [14].

### Laboratory tests

Blood was drawn after at least 12 h of fasting. Total cholesterol, high-density lipoprotein cholesterol (HDL-C) and triglycerides were measured enzymatically (Olympus^®^ AU640, Japan). Low-density lipoprotein cholesterol (LDL-C) was calculated according to Friedewald’s formula [15]. A 2-h oral glucose tolerance test (OGTT) was performed by measuring fasting plasma glucose and 2-h plasma glucose after ingestion of a glucose load of 75 g anhydrous glucose dissolved in water. Glucose values were measured from capillary whole blood with HemoCue^®^ Glucose 201+ system (Ängelholm, Sweden). The analyzer converts the result from capillary whole blood to plasma glucose (conversion factor 1.11).

### Appointment with the general practitioner

An appointment with the study GP was arranged for persons with high CVD risk (as described above) within 2–4 months after the nurse’s appointment. At that time, plasma lipids and fasting plasma glucose were retested, and an ECG and laboratory tests were collected to screen for secondary hypertension or dyslipidaemia. The GP examined the patients and gave lifestyle counseling. According to the national Finnish guidelines of that time, antihypertensive medication was prescribed if systolic BP was ≥160 mmHg or diastolic ≥100 mmHg. In patients with newly detected diabetes or hypertensive target organ damage (albuminuria, left ventricular hypertrophy on ECG), antihypertensive medication was initiated if systolic BP was ≥140 mmHg or diastolic BP ≥ 90 mmHg. If the study subjects had ongoing antihypertensive medication, it was intensified if systolic BP was ≥140 mmHg or diastolic ≥85 mmHg (≥80 mmHg in patients with diabetes. An antihypertensive drug, a lipid-lowering agent, or low-dose aspirin was prescribed if the 10-year risk for developing a fatal CVD event now or extrapolated to the age of 60 years was ≥5% estimated by the SCORE system [16]. A follow-up appointment with the study GP was arranged if new medications were started, or if previous medication was modified.

### Definitions and formation of study groups

**Fig. 1:**
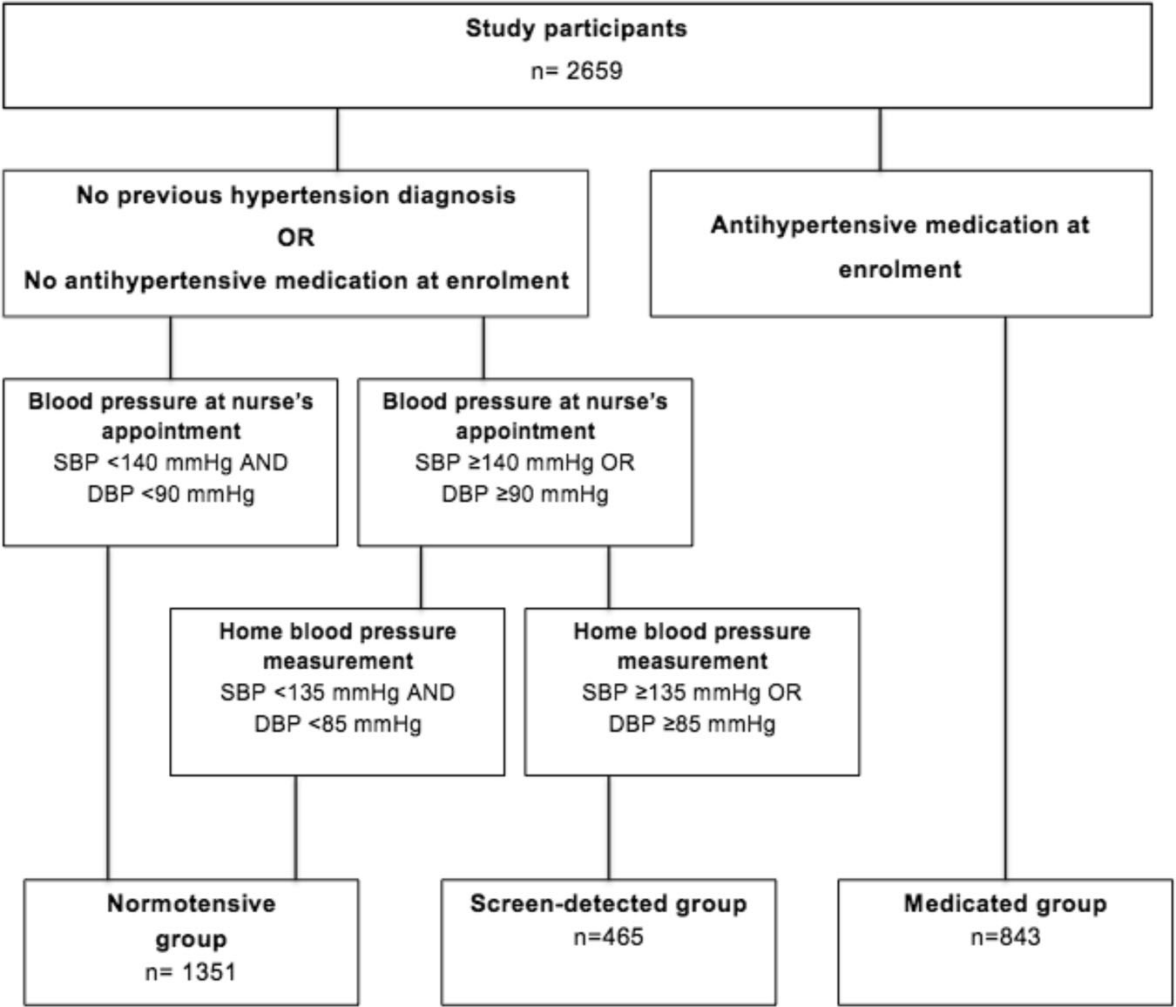
Formation of study groups.

The formation of study groups is illustrated in Fig. 1. Persons were defined as normotensives if they had no antihypertensive medication at enrollment, and the mean of nurse-measured BP was <140/90 mmHg or the average of home BP values was <135/85 mmHg. Screen-detected hypertension was diagnosed if the subject had no antihypertensive medication, and the mean of home BP measurements were ≥135 mmHg for systolic BP or ≥85 mmHg for diastolic BP [13]. Subjects having ongoing antihypertensive medication at enrollment were regarded as the medicated group.

Glucose metabolism disorders were categorized as T2D (fasting glucose ≥7.0 mmol/l or 2-h postload plasma glucose ≥12.2 mmol/l) [17]—those patients later referred as having newly diagnosed diabetes—and prediabetes including impaired glucose tolerance (IGT: 2-h postload plasma glucose 8.9–12.2 mmol/l), and impaired fasting glucose (IFG: 6.1-6.9 mmol/l).

LTPA level was categorized as low (LTPA for ≥30 min at a time for maximum of three times a week), moderate (LTPA for ≥30 min at a time for four to five times a week), and high (LTPA ≥ 30 min at a time for six or more times a week).

### Mortality

Data on mortality was obtained from Statistics Finland. Statistics Finland is an independently acting government agency belonging to the administrative sector of the Ministry of Finance. Statistics Finland has collected information on causes of death of Finnish citizens according to the International Classification of Diseases and Related Health Problems, 10th Revision (ICD-10) from the year 1996. We categorized the causes of death as deaths from all causes and deaths from cardiovascular causes: diseases of the circulatory system, I00-I99 (excluding I26 pulmonary embolism with acute cor pulmonale and I26.9 pulmonary embolism without acute cor pulmonale) and vascular dementia, F01.0-F01.9. For each person, the date of the invitation to the Harmonica project was the start date of the observational period. Follow-up time of mortality ended on December 31st, 2018.

### Statistical analyses

The descriptive statistics were presented as means with standard deviation (SD), as medians with interquartile range (IQR) or counts with percentages. Group differences in baseline were investigated through a series of one-way analysis of variances (ANOVA) and chi-square test with post hoc comparisons using Hommel’s correction. Kaplan-Meier’s survival analysis were performed to estimate cumulative all-cause and CVD mortality. Adjusted Kaplan-Meier cumulative mortality rates were estimated using two propensity score-based techniques, stratification and weighting (MMWS, marginal mean weighting through stratification) [18]. MMWS is an extension of propensity score matching that combines propensity score stratification and inverse probability of treatment weighting. Adjustments were made for age, gender, total cholesterol, newly diagnosed diabetes, education years, current smoking, LTPA, and BMI. We used Cox proportional hazards model to calculate the adjusted hazard ratios (HR) for death and the Fine and Gray competing risks regression model to calculate subhazard ratios (sHR) due to CVD mortality (with other causes death as a competing event). The proportional hazards assumption was tested graphically and by use of a statistical test based on the distribution of Schoenfeld residuals. The normality of variables was evaluated graphically and by using the Shapiro–Wilk W test. Stata 16.0 (StataCorp LP, College Station, TX, USA) was used for the statistical analyses.

## Results

The study population consisted of 2659 home-dwelling 45–70 years old subjects (55% women) without previous history of CVD or diabetes at baseline. Their mean age was 58 years (SD 7). There were 1351 (51%) normotensive subjects, 465 (17%) screen-detected hypertensives, and 843 (32%) medicated hypertensives. Table 1 shows the baseline characteristics of the subjects according to hypertension status.

**Table 1:** Baseline characteristics of the study participants.

	Hypertension status			*P* value^a^ [multiple comparison]
	Normotensive N = 1351	Screen-detected N = 465	Medicated N = 843	
Age, mean, years (SD)	57 (7)	58 (7)	60 (7)	<0.001 [N/S, N/M, S/M]
Females, n (%)	786 (58)	228 (49)	461 (55)	0.002 [N/S]
Education years, mean (SD)	10.7 (2.7)	10.3 (2.6)	9.9 (2.6)	<0.001 [N/S, N/M, S/M]
Body mass index, kg/m^2^, mean (SD)	27.5 (4.4)	28.7 (4.6)	31.0 (5.5)	<0.001 [N/S, N/M, S/M]
Waist circumference, cm, mean (SD)				
Women	88 (12)	92 (13)	98 (14)	<0.001 [N/S, N/M, S/M]
Men	99 (10)	101 (10)	106 (12)	<0.001 [N/S, N/M, S/M]
Current smoking, n (%)	247 (19)	86 (19)	130 (15)	0.14
AUDIT-score, mean (SD)	4.5 (4.7)	5.2 (5.3)	4.5 (4.9)	0.021 [N/S, N/M, S/M]
Leisure-time physical activity level, n (%)				0.015 [N/S, S/M]
Low	211 (16.1)	85 (18.8)	172 (21.0)	
Moderate	660 (50.4)	226 (49.9)	408 (49.8)	
High	438 (33.5)	142 (31.3)	240 (29.3)	
Blood pressure, mmHg, mean (SD)				
Systolic	132 (15)	157 (16)	144 (18)	<0.001 [N/S, N/M, S/M]
Diastolic	81 (8)	92 (10)	86 (10)	<0.001 [N/S, N/M, S/M]
Plasma lipids, mmol/l, mean (SD)				
Total cholesterol	5.41 (0.93)	5.53 (0.97)	5.27 (1.03)	<0.001 [N/S, N/M, S/M]
HDL cholesterol	1.61 (0.47)	1.56 (0.42)	1.44 (0.40)	<0.001 [N/S, N/M, S/M]
LDL cholesterol	3.26 (0.86)	3.35 (0.87)	3.15 (0.93)	<0.001 [N/M, S/M]
Triglycerides	1.29 (0.72)	1.42 (0.84)	1.54 (0.71)	<0.001 [N/S, N/M, S/M]
Plasma glucose, mmol/l, mean (SD)				
Fasting	5.50 (1.09)	5.59 (1.05)	5.84 (1.28)	<0.001 [N/M, S/M]
2h-glucose	6.92 (1.94)	7.47 (2.25)	8.20 (2.53)	<0.001 [N/S, N/M, S/M]
Glucose disorder, n (%)				<0.001 [N/S, N/M, S/M]
Prediabetes	151 (11)	68 (15)	161 (19)	
Type 2 diabetes	75 (6)	34 (7)	115 (14)	
Lipid-lowering medication, n (%)	72 (5)	36 (8)	227 (27)	<0.001 [N/M, S/M]

*AUDIT* Alcohol Use Disorders Identification Test.

^a^Hommel’s multiple comparison procedure was used to correct significance levels for post hoc testing *p* < 0.05.

The screen-detected hypertensives had higher mean systolic BP and diastolic BP values, total cholesterol level, and AUDIT scores than the two other groups. Compared to the normotensives, the screen-detected subjects were older, more often males, had higher BMI and larger WC, lower level of LTPA, higher 2-h plasma glucose concentration and higher amount of subjects with glucose disorders. The subjects in the medicated group had less optimal CVD risk profiles, e.g., older age, higher glucose levels and higher amount of subjects with newly diagnosed prediabetes and diabetes, higher BMI, larger WC, and higher triglyceride and lower HDL-C concentrations compared to the other groups. Persons in the medicated group also used more often lipid-lowering medication and were less educated compared to the two other groups.

Antihypertensive medication was initiated for 112 (24%) subjects in the screen-detected group. Lipid-lowering medication was prescribed for 107 (8%) subjects in the normotensive group, 91 (20%) in the screen-detected group, and 165 (20%) in the medicated group.

### Mortality

In the whole cohort, a total of 31,710 person-years were followed-up (median follow-up time 12.3 years). There were 289 (11%) deaths, 83 (29%) due to CVD. Unadjusted cumulative all-cause mortality over 13 years was 9.1% (95% CI: 7.6 to 10.8) in the normotensive, 9.9% (95% CI: 7.4 to 13.1) in the screen-detected, and 16.0% (95% CI: 13.6 to 18.8) in the medicated group (*p* < 0.001 log-rank test). Unadjusted cumulative CVD mortality over 13 years was 2.5% (95% CI: 1.8 to 3.5) in the normotensive, 1.8% (95% CI: 1.0 to 3.5) in the screen-detected, and 5.5% (95% CI: 4.1 to 7.3) in the medicated group (*p* < 0.001 log-rank test).

**Fig. 2:**
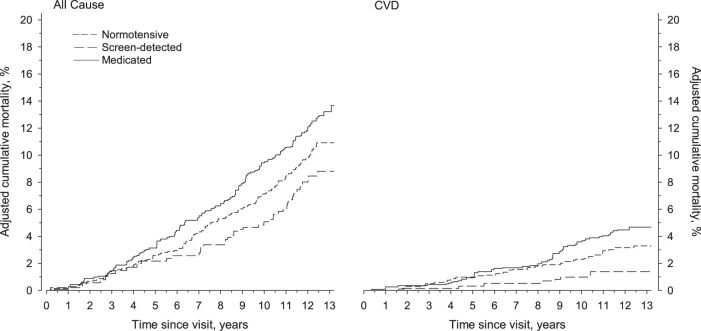
Adjusted cumulative all cause and cardiovascular (CVD) mortality. Adjustments were made for age, gender, total cholesterol, newly diagnosed diabetes, education years, smoking, leisure time, physical activity, and body mass index.

Adjusted cumulative all-cause and CVD mortality according to hypertension status is illustrated in Fig. 2. When adjusted for age, gender, total cholesterol, newly diagnosed T2D, education years, current smoking, LTPA, and BMI, there was no statistically significant difference in all-cause mortality rates between the study groups (*p* = 0.053). Regarding adjusted CVD mortality rates the difference between the study groups was significant (*p* = 0.012).

The most common cardiovascular cause of death was atherosclerotic heart disease (ICD-10 code I25.1), which caused 30% of all cardiovascular deaths.

Table 2 shows competing risk regression model for the relationship between CVD risk factors and CVD mortality. When compared to the medicated group, those with screen-detected hypertension had decreased CVD mortality risk: sHR 0.40 (95% CI: 0.19 to 0.88), *p* = 0.023. CVD mortality risk in the screen-detected group was statistically comparable to the normotensive group: HR 0.53 (0.24 to 1.15), *p* = 0.11. The other independent predictors of CVD mortality were older age, male gender, newly diagnosed diabetes, and smoking.

**Table 2:** Competing risk regression (Fine and Gray hazards model) for the relationship between cardiovascular disease (CVD) risk factors and CVD mortality.

	CVD mortality	
	sHR^a^ (95%CI)	*p* value
Hypertension status		
Medicated	1.00 (Reference)	
Normotensive	0.77 (0.45 to 1.31)	0.33
Screen-detected	0.40 (0.19 to 0.88)	0.023
Age	1.12 (1.07 to 1.17)	<0.001
Male gender	2.57 (1.60 to 4.11)	<0.001
Body mass index	1.00 (0.95 to 1.05)	0.95
Total cholesterol	0.97 (0.73 to 1.29)	0.84
Newly diagnosed diabetes	2.71 (1.57 to 4.69)	<0.001
Education years	0.95 (0.86 to 1.06)	0.38
Smoking	1.81 (1.08 to 3.03)	0.025
Leisure-time physical activity level		
Low	1.00 (Reference)	*P* for linearity = 0.75
Moderate	1.04 (0.56 to 1.92)	
High	1.11 (0.57 to 2.13)	

^a^Subhazard ratio, competing-risks regression model was used where the rest of the causes of death were considered as competing risks.

## Discussion

In this community-based study of subjects at risk but without manifested CVD or diabetes, previously undiagnosed hypertension was detected in 17% of the participants. After an intervention feasible in primary care settings, the long-term CVD mortality risk of the screen-detected hypertensives was comparable to the level of normotensive CVD risk persons. The CVD mortality risk of the medicated hypertensives at baseline was more than two-fold compared to the subjects with screen-detected hypertension.

The study was conducted among a population typical in primary care setting. Every other of the identified CVD risk persons had confirmed hypertension but one in three were unaware of it. Previous multinational BP screening studies also show low levels of awareness (up to 50%) and inadequate control of hypertension [7, 19], even in high-income countries. However, these studies lack the confirmation of hypertension diagnosis with out-of-office BP measurements. In Finland, the overall prevalence of hypertension is estimated to be over 50% in the adult population, only 50% of the hypertensive subjects use antihypertensive medication, and less than half of them reach the treatment target, even though hypertension care has improved significantly in Finland during the last decades [20].

In our study population, the CVD mortality rate was highest in the previously medicated group with suboptimal mean systolic and diastolic BP levels. The control of both diastolic BP and systolic BP have been found essential for reducing CVD mortality when compared to normotensive people [21]. Previous studies have also suggested a residual cardiovascular risk in BP-medicated individuals, e.g., the normalization of BP by antihypertensive medication does not neutralize the CVD risk or subclinical disease burden of the individuals [22, 23].

Furthermore, CVD mortality risk is associated with other co-existing CVD risk factors, too. In our analyses, the observed CVD risk factors predicting CVD mortality were previously well established, i.e., “classical risk factors”. However, the impact of newly diagnosed T2D was surprisingly high. Our study population consisted of subjects with at least one CVD risk factor at baseline, with the medicated hypertensives having the worst risk factor profile and the worst prognosis. The multifactorial intervention performed by a nurse and a GP was associated with better results among the newly diagnosed than the previously diagnosed hypertensives. It is possible that the medicated subjects already had more progressed subclinical atherosclerosis contributing to worse prognosis.

In previous prospective studies without multifactorial intervention, the prognosis of newly diagnosed hypertensives has been worse than in normotensive subjects. A follow-up study of the population-based cohorts of the national Finnish health surveys in 1972–1997, showed that individuals with newly diagnosed and previously diagnosed hypertension at baseline have a higher risk of all-cause and CVD mortality than normotensive individuals [21]. The post-hoc observational study of the Antihypertensive and Lipid-Lowering Treatment to Prevent Heart Attack Trial (ALLHAT) found no statistically significant difference between treatment-naïve and previously treated hypertensive participants in all-cause or CVD mortality [24]. However, in the ALLHAT study, the median time of the in-trial period was only five years. Similar to our study, the subjects with antihypertensive medication at baseline were more likely to have lower BP and a lower rate of smoking, but they had higher estimated baseline risk due to the higher proportion of subjects having diabetes [24].

The main limitation of the present study is the lack of randomization. Also, because the purpose was to study if population screening and early detection of hypertension had any significance in the long run, the measurements and CVD risk factor evaluations were made only at baseline. Thus, we have no information on BP values during the follow-up or the study subjects’ compliance to medical treatment. All subjects were free to use the common healthcare providers in the community as normal; prior, during, and after the study. It is possible that during the follow-up time the persons who were normotensive at baseline could become hypertensive and develop more CVD risk factors. This might explain why a trend towards increased mortality was observed in the normotensive group in comparison with the screen-detected group. The findings in this study support the European Society of Hypertension (ESH)/European Society of Cardiology (ESC) guidelines which recommend BP-screening at regular intervals also for healthy normotensive people every 1 to 5 years, depending on the BP level [25].

Strengths of the present study include the community-based representative sample of apparently healthy subjects at risk for CVD. Also, the intervention used was typical for general practice, where most patients with hypertension are treated and screened for. The in-office measurements were made by trained medical staff, and the participants’ BP status was verified by home measurements of BP. Thus, bias regarding white-coat hypertension was excluded. The follow-up time of 13 years was long enough to accumulate a sufficient number of outcome events. Furthermore, the causes of death were obtained from national registers that have high validity in Finland.

In conclusion, undiagnosed hypertension is common in primary care population. Targeted screening, lifestyle counseling, and prescription of evidence-based medication were associated with long-term CVD mortality risk among screen-detected hypertensive individuals comparable to that of normotensive CVD-risk persons at baseline. This study emphasizes the importance of early detection of hypertension and multifactorial intervention in a CVD-risk population.

## Summary

### What is known about topic

- In community-level, hypertension remains commonly undetected and undermedicated.
- Both high-risk and population-based strategies have been recommended to improve hypertension management.
- Evidence about the effectiveness of screening strategies for reducing hypertension-related morbidity and mortality is scarce.

### What this study adds

- Screening for hypertension with home blood pressure monitoring is quite easy in primary care setting.
- Timely multifactorial intervention seems to be effective in preventing hypertension-related mortality.

## Data Availability

Additional data are available from the corresponding author on reasonable request.

## References

[1] Forouzanfar MH, Liu P, Roth GA, Ng M, Biryukov S, Marczak L (2017). Global burden of hypertension and systolic blood pressure of at least 110 to 115 mmHg, 1990-2015. J Am Med Assoc.

[2] GBD 2015 Risk Factors Collaborators. (2016). Global, regional, and national comparative risk assessment of 79 behavioural, environmental and occupational, and metabolic risks or clusters of risks, 1990–2015: a systematic analysis for the Global Burden of Disease Study 2015. Lancet.

[3] Haldar RN. Global Brief on Hypertension: Silent Killer, Global Public Health Crisis [Internet]. World Health Organization. 2013 [accessed 30 Jan 2020]. Available from: http://apps.who.int/iris/bitstream/10665/79059/1/WHO_DCO_WHD_2013.2_eng.pdf.

[4] Brunström M, Carlberg B (2018). Association of blood pressure lowering with mortality and cardiovascular disease across blood pressure levels: a systematic review and meta-analysis. JAMA Intern Med.

[5] Ettehad D, Emdin CA, Kiran A, Anderson SG, Callender T, Emberson J (2016). Blood pressure lowering for prevention of cardiovascular disease and death: a systematic review and meta-analysis. Lancet [Internet].

[6] Zanchetti A, Thomopoulos C, Parati G (2015). Randomized controlled trials of blood pressure lowering in hypertension a critical reappraisal. Circulation.

[7] Beaney T, Schutte AE, Tomaszewski M, Ariti C, Burrell LM, Castillo RR (2018). May Measurement Month 2017: an analysis of blood pressure screening results worldwide. Lancet Glob Heal.

[8] Adler AJ, Prabhakaran D, Bovet P, Kazi DS, Mancia G, Mungal-Singh V (2015). Reducing cardiovascular mortality through prevention and management of raised blood pressure: a World Heart Federation roadmap. Glob Heart.

[9] Schmidt B-M, Durao S, Toews I, Bavuma CM, Hohlfeld A, Meerpohl J (2020). Screening strategies for hypertension (Review). Cochrane Database Syst Rev.

[10] Korhonen PE, Jaatinen PT, Aarnio PT, Kantola IM, Saaresranta T (2009). Waist circumference home measurement—a device to find out patients in cardiovascular risk. Eur J Public Health.

[11] Lindström J, Tuomilehto J (2003). The diabetes risk score. Diabetes Care [Internet].

[12] Babor T, de la Fuente J, Saunders J, Grant M. AUDIT: the alcohol use disorders identification test: guidelines for use in primary healthcare. WHO/MNH/DAT 89.4, Geneva: World Health Organization; 1989.

[13] Parati G, Stergiou GS, Asmar R, Bilo G, De Leeuw P, Imai Y (2008). European society of hypertension guidelines for blood pressure monitoring at home: A summary report of the second international consensus conference on home blood pressure monitoring. J Hypertens.

[14] Alberti K, Zimmet P, Shaw J, Consensus Group IETF (2005). The metabolic syndrome—a new worldwide definition. Lancet.

[15] Friedewald WT, Levy RI, Fredrickson DS (1972). Estimation of the concentration of low-density lipoprotein cholesterol in plasma, without use of the preparative ultracentrifuge. Clin Chem.

[16] Conroy RM, Pyörälä K, Fitzgerald AP, Sans S, Menotti A, De Backer G (2003). Estimation of ten-year risk of fatal cardiovascular disease in Europe: the SCORE project. Eur Heart J.

[17] Definition and diagnosis of diabetes mellitus and intermediate hyperglycemia [Internet]. Report of a WHO/IDF consultation. 2006. http://care.diabetesjournals.org/content/26/3/725.short.

[18] Linden A (2014). Combining propensity score-based stratification and weighting to improve causal inference in the evaluation of health care intervention. J Eval Clin Pract.

[19] Chow C, Teo K, Rangarajan S, Islam S, Gupta R, Avezum A (2013). Prevalence, awareness, treatment, and control of hypertension in rural and urban communities in high-, middle-, and low-income countries. J Am Med Assoc.

[20] Koponen P, Borodulin K, Lundqvist A, Sääksjärvi K, Koskinen S. Health, functional capacity and welfare in Finland—FinHealth 2017 study. (abstract in English) [Internet]. National Institute for Health and Welfare (THL). 2018 [accessed 19 Nov 2019]. http://urn.fi/URN:ISBN:978-952-343-105-8.

[21] Barengo NC, Kastarinen M, Antikainen R, Nissinen A, Tuomilehto J (2009). The effects of awareness, treatment and control of hypertension on cardiovascular and all-cause mortality in a community-based population. J Hum Hypertens.

[22] Lieb W, Enserro DM, Sullivan LM, Vasan RS (2015). Residual cardiovascular risk in individuals on blood pressure-lowering treatment. J Am Heart Assoc.

[23] Blacher J, Evans A, Arveiler D, Amouyel P, Ferrières J, Bingham A (2010). Residual cardiovascular risk in treated hypertension and hyperlipidaemia: the PRIME study. J Hum Hypertens.

[24] Ho CLB, Breslin M, Chowdhury EK, Doustd J, Reid CM, Davis BR (2020). Lack of a significant legacy effect of baseline blood pressure “treatment naivety” on all-cause and cardiovascularmortality in the antihypertensive and lipid-lowering treatment to prevent heart attack trial. J Hypertens.

[25] Williams B, Mancia G, Spiering W, Agabiti Rosei E, Azizi M, Burnier M (2018). 2018 ESC/ESH Guidelines for the management of arterial hypertension: the Task Force for the management of arterial hypertension of the European Society of Cardiology (ESC) and the European Society of Hypertension (ESH). Eur Heart J.

